# Accumulation of 2-hydroxyglutarate in gliomas correlates with survival: a study by 3.0-tesla magnetic resonance spectroscopy

**DOI:** 10.1186/s40478-014-0158-y

**Published:** 2014-11-07

**Authors:** Manabu Natsumeda, Hironaka Igarashi, Toshiharu Nomura, Ryosuke Ogura, Yoshihiro Tsukamoto, Tsutomu Kobayashi, Hiroshi Aoki, Kouichirou Okamoto, Akiyoshi Kakita, Hitoshi Takahashi, Tsutomu Nakada, Yukihiko Fujii

**Affiliations:** Department of Neurosurgery, Brain Research Institute, University of Niigata, Niigata, Japan; Center for Integrated Brain Sciences, Brain Research Institute, University of Niigata, Niigata, Japan; Department of Pathology, Brain Research Institute, University of Niigata, Niigata, Japan

**Keywords:** Glioma, MRS, 2-hydroxyglutarate, *IDH* mutation, Prognostic marker

## Abstract

**Introduction:**

Previous magnetic resonance spectroscopy (MRS) and mass spectroscopy studies have shown accumulation of 2-hydroxyglutarate (2HG) in mutant isocitrate dehydrogenase (*IDH*) gliomas. *IDH* mutation is known to be a powerful positive prognostic marker in malignant gliomas. Hence, 2HG accumulation in gliomas was assumed to be a positive prognostic factor in gliomas, but this has not yet been proven. Here, we analyzed 52 patients harboring World Health Organization (WHO) grade II and III gliomas utilizing 3.0-tesla MRS.

**Results:**

Mutant IDH gliomas showed significantly higher accumulation of 2HG (median 5.077 vs. 0.000, *p* =0.0002, Mann–Whitney test). 2HG was detectable in all mutant *IDH* gliomas, whereas in 10 out of 27 (37.0%) wild-type *IDH* gliomas, 2HG was below the detectable range (2HG =0) (*p* =0.0003, chi-squared test). Screening for *IDH* mutation by 2HG analysis was highly sensitive (cutoff 2HG =1.489 mM, sensitivity 100.0%, specificity 72.2%). Gliomas with high 2HG accumulation had better overall survival than gliomas with low 2HG accumulation (*p* =0.0401, Kaplan-Meier analysis).

**Discussion:**

2HG accumulation detected by 3.0-tesla MRS not only correlates well with *IDH* status, but also positively correlates with survival in WHO grade II and III gliomas.

**Electronic supplementary material:**

The online version of this article (doi:10.1186/s40478-014-0158-y) contains supplementary material, which is available to authorized users.

## Introduction

**Figure 1:**
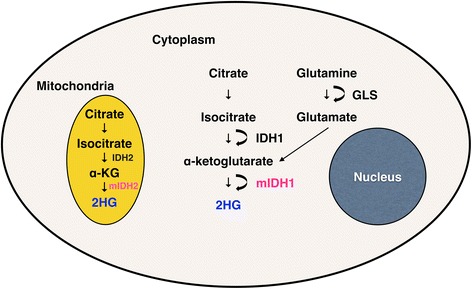
**Schematic representation of 2HG production in** ***IDH*** **mutant gliomas.** Accumulation of 2HG is seen in the cytoplasm of mutant *IDH1* and mitochondria of mutant IDH2 gliomas. 2HG is also derived from glutamine in mutant IDH gliomas.

A comprehensive genomic analysis of glioblastomas has shown that mutations of isocitrate dehydrogenase (*IDH*) are found in a subset of glioblastoma [1], and subsequent studies have found *IDH* mutation to be a powerful prognostic factor in malignant gliomas [2], suggesting that *IDH* mutations represent a clinically distinct subset of gliomas. The accumulation of 2-hydroxyglutarate (2HG) is noted in the cytoplasm of glioma cells with *IDH1* mutation and in the mitochondria of cells with *IDH2* mutation (Figure 1) [3]. Magnetic resonance spectroscopy (MRS) [4-10] as well as mass spectrometry [3,10-12] are known to effectively measure 2HG in glioma tissues with good correlations to *IDH* mutation status. 2HG is an oncometabolite, which has been shown to cause tumorigenesis by inhibition of histone demethylation [13-15] and DNA demethylation [16,15]. 2HG accumulation in gliomas was assumed to positively correlate with patient survival because of the correlation of *IDH* status to patient survival in malignant gliomas. However, to date, this has not been proven. In the present study, 2HG accumulation was shown to have a positive correlation with overall patient survival in WHO grade II and III gliomas for the first time.

## Materials and methods

### Participants

Seventy-one adult patients harboring World Health Organization (WHO) grade II or III gliomas, receiving magnetic resonance spectroscopy (MRS) evaluation at the Center for Integrated Brain Science, University of Niigata, before surgery and surgical treatment at the Department of Neurosurgery, University of Niigata, from December 2006 to March 2013 were included in the study. Patients with non-astrocytic, non-oligodendroglial, and non-oligoastrocytic tumors (e.g. ependymomas, n =11), patients whose MRS scans had low signal-to-noise ratios (S/N) of less than 4 (n =4), patients having a glioblastoma-like single voxel MRS (SVMRS) spectra at relapse reflecting radiation necrosis or malignant transformation (n =2), a patient harboring a cystic lesion with insufficient volume of a solid component (n =1), and a patient lost to follow up (n =1), were excluded from the analysis. Thus, a total of 52 patients were ultimately analyzed. Written informed consent was obtained from all of the participants in accordance with the human research guidelines of the Internal Review Board of University of Niigata.

### MRS analysis

MRI/^1^H-MRS was performed using a 3.0-tesla system (Signa LX, General Electric, Waukesha, WI) with an 8 channel phased array coil head. First, proton density images (Fast Spin Echo; TR/TE =5000/40; FOV: 20 × 20 mm; matrix: 256 × 256; slice thickness: 5 mm; inter slice gap: 2.5 mm) were taken. The slice with the largest depiction of tumor on proton density images was selected for SVMRS. A point-resolved spectroscopic sequence (PRESS), with chemical-shift-selective water suppression was used with the following parameters: (TR: 1.5 s; TE: 30 ms; data point 512; spectral width 1000Hz; number of acquisitions: 128–196; volume of interest (VOI): 12–20 × 12–20 × 12–20 mm).

Spectral analysis was performed using LCModel version 6.3 (Stephen Provencher, Oakville, Ontario, Canada) [17]. This software automatically adjusts the phase and chemical shift of the spectra, estimates the baseline, and performs eddy current corrections. Relative metabolite concentrations and their uncertainties were estimated by fitting the spectrum to a basis set of spectra acquired from individual metabolites in solution. The basis set was made with MR experiment simulation software (GAMMA, Radiology, Duke University Medical Center, Durham, NC) and provided by Dr.Steven Provencher [17] and was calibrated with MRS phantom solution (18-cm-diameter MRS HDsphere, model 2152220; General Electric, Milwaukee, WI) using our MR system. Nineteen metabolites were included in this LCModel basis set: alanine, aspartate, creatine (Cr), phosphocreatine (PCr), γ- aminobutyric acid, glucose, glutamine (Gln), glutamate (Glu), glycerophosphocholine (GPC), phosphocholine (PC), gluthathione (GSH), 2-hydroxyglutarate (2HG), myo-inositol (Ins), lactate, NAA (N-acetylaspartate), N-acetylaspartylglutamate (NAAG), scyllo-inositol, taurine, and guanine. Total NAA (tNAA: the sum of NAA and NAAG), total choline (tCho: the sum of GPC and PC), total creatine (tCr: the sum of Cr and PCr), and sum of Glu and Gln (Glx) were noted. To calculate the absolute metabolite concentrations, an unsuppressed water signal was used as a reference.

Quantification estimates of metabolites were considered unreliable and excluded when Cramer-Rao lower bounds, returned as the percentage of standard deviation (%SD) by LCModel, was greater than 35%, as previously described [18]. Because low 2HG and GSH estimates yielded large %SDs (i.e. when 2HG =0, %SD = ∞), the above exclusion criteria was applied only when the estimated 2HG amount was greater than 1.0 mM or GSH was greater than 0.5 mM. Glx and tNAA were excluded when %SD was greater than 30%; tCho and tCr were excluded when %SD was greater than 20%.

### Pathological analysis and IDH analysis

Surgical specimens were analyzed by two pathologists (H.T. and A.K.) and diagnosed according to the WHO classification [19]. IDH1 R132H immunohistochemical (IHC) analysis (H09 clone, Dianova, Hamburg, Germany; 1:100) was performed in formalin-fixed, paraffin imbedded section using the avidin-biotin-peroxide method (Vector, Burlingame, CA, USA) with diaminobenzidine as the chromogen and counterstained with hematoxylin.

For cases showing negative staining for IDH1 R132H, DNA sequencing for *IDH1* and *IDH2* was analyzed. Genomic DNA was extracted from paraffin-embedded sections, and as described previously [20,21], PCR amplification was performed by using primer sets (forward: 5’-CGGTCTTCAGAGAAGCCATT-3’ , and reverse 5’-TTCATACCTTGCTTAATGGGTGT-3’) at codon 132 for the *IDH1* gene and (forward: 5’-AATTTTAGGACCCCCGTCTG-3’ , and reverse 5’-CTGCAGAGACAAGAGGATGG-3’) at codon 172 for the *IDH2* gene. The PCR products were then sequenced on a 3130xl Genetic Analyzer (Applied Biosystems, Foster City, CA, USA) with a Big Dye Terminator v1.1 Cycle Sequencing Kit (Applied Biosystems) in accordance with the manufacturer’s instructions.

### Statistical analysis

Corrected metabolite concentrations of patients harboring mutant *IDH* gliomas were compared to those harboring gliomas of wild-type *IDH* using the Mann–Whitney U test. Receiver operating characteristic (ROC) curve was used to determine a cutoff for 2HG concentration to obtain maximal sensitivity and specificity to identify *IDH* mutations. Kaplan-Meier analysis was used to compare overall survival. Tests for associations between different parameters were carried out by the chi-squared test for 2 × 2 contingency tables. *p* <0.05 was considered significant. Statistical analyses were performed using GraphPad Prism 6 software (GraphPad Software, http://www.graphpad.com).

## Results

A summary of the patient characteristics of mutant and wild-type IDH groups is provided in Table 1. Median patient age was 53 years; Patients harboring mutant *IDH* gliomas were younger than those with wild-type *IDH* gliomas (45 years vs. 61 years, *p* =0.0008, Mann–Whitney U test). A majority (90.4%) of the patients analyzed were newly-diagnosed patients. *IDH* mutations were found in only 25 out of 52 cases (48.1%), this was probably due to: the inclusion of primary glioblastoma and glioblastoma with oligodendroglioma component, failure to detect rare *IDH1* and *IDH2* mutations by DNA sequencing, and/or selection bias due to the preoperative availability of MRS. There were more WHO grade II tumors (68.0% vs. 25.9%, *p* =0.0024) and more patients were alive at last follow-up in the mutant IDH group (80.0% vs. 44.4%, *p* =0.0085).

**Table 1:** **Patient characteristics of mutant and wild-type** ***IDH*** **groups**

**Characteristic**	**Number of patients (%)**		***p*** **value**
	**Mutant** ***IDH***	**Wild-type** ***IDH***	
Number	25	27	
Men: Women	14:11	14:13	
Age (years)			
Median	45	61	0.0008*
Range	26-67	28-77	
Newly diagnosed	22 (88.0)	25 (92.6)	0.9279
Recurrent	3 (12.0)	2 (7.4)	
Pathological grade			
WHO Grade II	17 (68.0)	7 (25.9)	0.0024*
WHO Grade III	8 (32.0)	20 (74.1)	
Outcome			
Alive	20 (80.0)	12 (44.4)	0.0085*
Dead	5 (20.0)	15 (55.6)	

Results of unpaired t-test (age) and chi-squared tests (others). The values inside parentheses represent percentage of patients within each group.

*p <0.05.

IDH: isocitrate dehydrogenase; WHO: World Health Organization.

**Figure 2:**
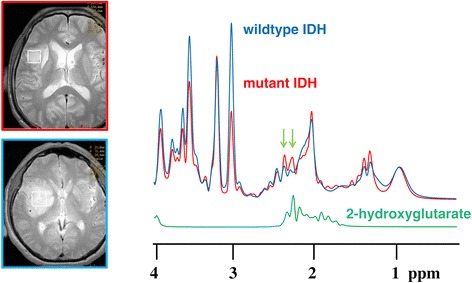
**SVMRS spectra of mutant** ***IDH*** **and wild-type** ***IDH*** **gliomas.** Representative SVMRS spectra of mutant *IDH* (red) and wild-type *IDH* gliomas (blue) are shown. Small peaks were detected at a chemical shift of about 2.25 ppm in mutant *IDH* gliomas. Both spectra have similar choline peaks, but these were not adjusted for choline.

Representative SVMRS spectra of mutant *IDH* and wild-type *IDH* gliomas are provided in Figure 2. Small peaks were detected at a chemical shift of about 2.25 ppm in mutant *IDH* gliomas. Both spectra have similar choline peaks, but these were not adjusted for choline.

**Figure 3:**
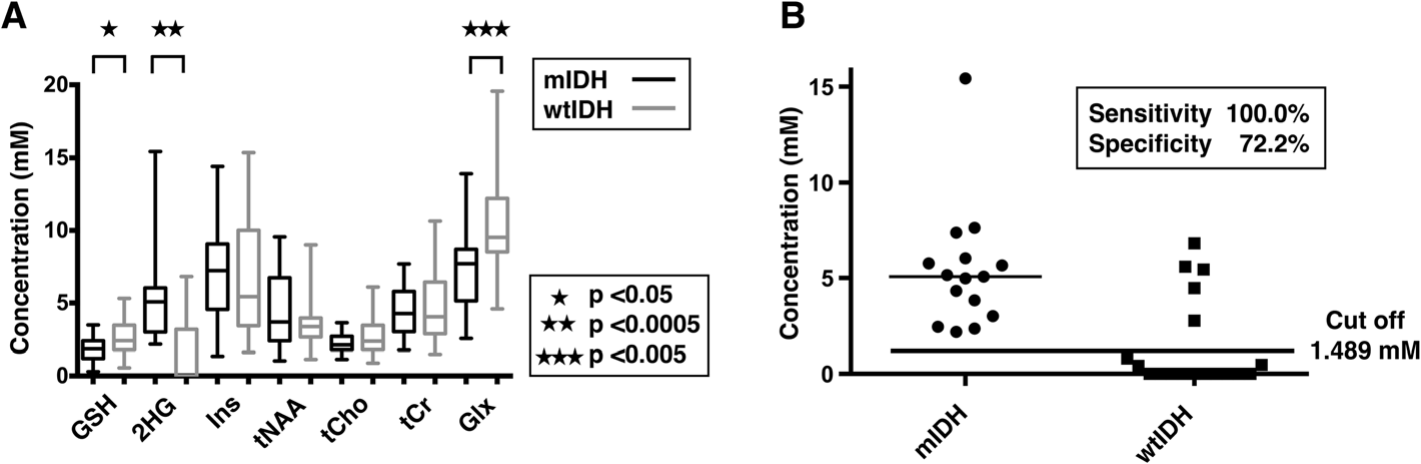
**2HG is accumulated in mutant** ***IDH*** **gliomas. A)** Comparisons of amount of metabolites in mutant *IDH* and wild-type *IDH* gliomas show markedly higher accumulation of 2-HG (median 5.007 mM vs. 0.000 mM, Mann–Whitney test, *p* =0.0002) and lower concentrations of Glx (*p* <0.05) in mutant *IDH* gliomas. **B)** ROC curve analysis revealed an optimal cutoff of 1.489, with a sensitivity of 100.0% and specificity of 72.2%. 2HG was detectable in all mutant *IDH* gliomas, whereas in 10 out of 27 (37.0%) wild-type *IDH* gliomas, 2HG was not detectable (2HG =0) (*p* =0.0003, chi-squared test). Five out of 27 (10.3%) wild-type *IDH* gliomas yielded a 2-HG concentration higher than 1.489 mM.

Mutant *IDH* gliomas showed a significantly higher accumulation of 2HG (median 5.077 mM vs. 0.000 mM, *p* =0.0002, Mann–Whitney test). Mutant *IDH* gliomas also showed lower levels of GSH (median 1.849 vs. 2.409, p =0.0328) and Glx (median 7.701 vs. 9.528, p =0.001) compared to the wild-type IDH gliomas (Figure 3A). Levels of Ins, tNAA, tCho, and tCr were not significantly different between the two groups.

**Figure 4:**
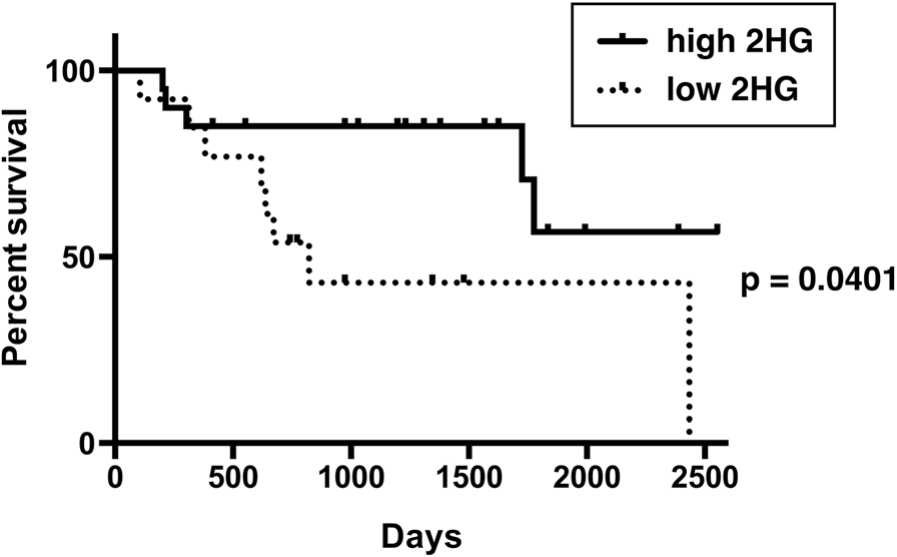
**Longer overall survival in high 2HG glioma patients.** The overall survival was significantly longer in glioma patients with high accumulation of 2HG (2HG >1.489) compared with low accumulation (*p* =0.401, Kaplan-Meier analysis).

ROC curve analysis obtained a cutoff of 2HG =1.489 mM, with a sensitivity of 100.0% and specificity of 72.2%, to detect *IDH* mutations (Figure 3B). 2HG was detectable in all mutant *IDH* gliomas, whereas in 10 out of 27 (37.0%) wild-type *IDH* gliomas, 2HG was not detectable (2HG =0) (*p* =0.0003). Five (18.5%) of the wild-type *IDH* gliomas had an accumulation of 2HG higher than 1.489 mM; three gliomas (11.1%) yielded a concentration of 2HG higher than 5 mM (Figure 3B). A significantly longer overall patient survival was noted in gliomas with high accumulation of 2HG (*p* =0.0401, Figure 4). Median survival was 823 days in glioma patients with low 2HG; median patient survival was not reached in the glioma patients with high 2HG. There was no significant difference in survival between patients harboring wild-type *IDH* glioma patients with high 2HG accumulation (2HG >1.489 mM) vs. low 2HG accumulation (p =0.4894, Kaplan-Meier curves not shown). Likewise there was no significant difference in survival between mutant *IDH* gliomas with high 2HG accumulation (2HG >5.077 mM) vs. low 2HG accumulation (p =0.8815, Additional file 1: Figure S1), although median survival has not been reached in either group.

## Discussion

IDH1 and IDH2 enzymes catalyze oxidative decarboxylation of isocitrate to α-ketoglutarate (α-KG). Mutant IDH cannot catalyze this reaction and instead reduces α-KG to 2HG [3] (Figure 1). 2HG is oxidized by 2-hydroxyglutarate dehydrogenase (2-HGDH) back to α-KG, and the mutation of 2-HGDH is known to cause 2-hydroxyglutaric aciduria [22]. A previous study has shown that glutamate is the main source of carbons for 2HG in mutant *IDH* glioma cells [3].

In our study, 2HG was detected in all mutant *IDH* gliomas. On the other hand, in a subset of wild-type *IDH* gliomas, a high 2HG concentration was noted (Figure 3B). This may be attributed to false-positive results [23] or a failure to detect rare *IDH1* or *IDH2* mutations by DNA sequencing. However, a recent study showed millimolar concentrations of 2HG in wild-type *IDH* breast cancer tissues. These accumulations were found to be associated with MYC, and carry a poor prognosis [24]. It remains to be seen if mechanisms of 2HG accumulation unrelated to *IDH* mutation exist in gliomas as well.

It is known that 2HG is primarily derived from glutamine in mutant *IDH* gliomas. Glutamine is hydrolyzed by glutaminase to produce glutamate, which is subsequently converted to α-KG [3,25]. MYC is known to regulate glutamine utilization and glutaminase protein expression [26], and mutant *IDH* gliomas are known to have an increased expression of MYC [27]. Interestingly, we found less accumulation of Glx (Glu + Gln) in the mutant *IDH* gliomas (*p* <0.005, Figure 3), suggesting that glutamine consumption is contributing to the accumulation of α-KG and ultimately 2HG (Figure 1) in these tumors.

2HG acts as a competitive antagonist of α-KG, causing inhibition of α-KG-dependent dioxygenases. These include the JmjC domain-containing histone demethylases (KDMs), which cause histone demethylation [13-15], and the ten-eleven translocation (TET) family of DNA hydroxylases, which cause DNA demethylation [16,15]. This was consistent with data from the The Cancer Genome Atlas (TCGA) database, in which the proneural subgroup of glioblastoma was found to be enriched with *IDH* mutations and display hypermethylation in a large number of loci [28]. A recent report has shown the stimulation of HIF prolyl hydroxylases by (R) enantiomer of 2HG in mutant *IDH* immortalized astrocytes leads to a reduced level of HIF, but enhanced proliferation [29].

There are still others who hypothesize that mutant IDH is not tumorigenic, but actually makes tumor cells susceptible to death, evidenced by the longer survival of patients with IDH mutant glioma patients [30]. Mutant IDH1 and 2HG were shown to induce oxidative stress, cell-killing autophagy and apoptosis in a cell type specific manner [31]. New evidence suggests that IDH1 mutation inhibits the growth of glioma cells via GSH inhibition and generation of reactive oxygen species (ROS) [32]. This study, as well as previous MRS [10] and metabolomic [11] studies have shown that GSH is depleted in mutant IDH gliomas.

At least 8 different mutations of *IDH1* and *IDH2* are known in gliomas, at the *IDH1* R132 and *IDH2* R172 loci. 2HG can be detected in gliomas *in vitro* by mass-spectrometry or *in vivo* by MRS. One of the proposed advantages of detecting 2HG is that it would provide a screening for all mutations of *IDH1* and *IDH2,* as all *IDH* mutations that are known to produce 2HG [33].

The 2HG molecule contains five nonexchangeable protons, giving rise to multiplets at three locations on 3 T MRS: approximately 4.02, 2.25, and 1.90 ppm (Figure 2) [5]. The multiplet at 2.25 ppm is larger than the other 2HG multiplets. The detection of this multiplet is complicated by the spectral overlap of Glu (2.43 ppm), Gln (2.34 ppm), and GABA (2.28 ppm) [34]. Direct detection of the multiplet at 1.90 ppm is difficult due to its proximity to NAA resonance at 2.01 ppm. Finally, the multiplet at 4.02 is partially overlapped with Cr (3.92 ppm), PCr (3.94 ppm), Ins (4.06 ppm), lactate (4.1 ppm) and free Cho (4.05 ppm) [5].

A false-positive rate of approximately 22% was observed by Pope et al. using the short-echo MRS with TE at 30 ms for the detection of 2HG [10]. This false-positive rate can be reduced by using long-echo MRS with TE at 97 ms with the use of three-dimensional volume-localized basis (VLB) spectra, which has been shown to be optimal for detection of 2HG [5,6]. A comparative study of PRESS sequences at short- (35 ms) and long- TE (97 ms) found long- TE to be superior for the following reasons: 1) it permits a more favorable voxel localization, and 2) it produces a well-defined narrow 2HG signal at 2.25 ppm, thereby leading to improved differentiation between 2HG and Glu, Gln, and GABA signals. Spectral fitting of PRESS data at TE =97 ms was effective in minimizing the effect of macromolecule signals [5]. Five (18.5%) wild-type IDH gliomas in this study were found to have high 2HG accumulation of more than 1.489 mM. Further analysis of these specimens by either mass spectrometry or *ex vivo* MRS is needed to determine whether these results could be attributed to false positive readouts.

Unambiguous detection of 2HG in mutant IDH glioma was achieved by 2D correlation spectroscopy (COSY) [4,7,8] and J-difference spectroscopy [4]. However, these methods are less available clinically and involve longer acquisition time; 2D correlation MRS involves complex quantification and has less sensitivity [23]. We achieved 100% sensitivity of 2HG detection by short-echo MRS with modulation of 2HG resonances by spectral fitting. Less acquisition time enabled glioma patients, even those with relatively poor performance status, to undergo analysis. The biggest advantage of detecting 2HG by MRS is that it provides an opportunity for pre-surgical, non-invasive detection of 2HG, thus reliably predicting *IDH* status of gliomas before surgery. There is increasing interest that mutant *IDH* patients may benefit from extensive surgery [35,36]. Also, 2HG is known to degrade after formalin fixation and paraffin embedding [12]. *Ex vivo* assessment of 2HG by MRS or mass spectrometry enable the analysis of homogeneous tumor tissue, but sample degradation and the necessity for treating tissues with reagents pose problems [23].

2HG detection by *in vivo* MRS may be utilized to evaluate response to glioma treatments. *IDH* mutations are known to be very tumor-cell-specific [37], and 2HG accumulation is found to be increased in tumor tissues compared to surrounding tissue. This leads to the notion that 2HG will not be assessable after surgical removal of a majority of the tumor. However, gliomas are pathologically known to be very infiltrative tumors, with individual glioma cells extending deep into adjacent brain tissues [38]. If 2HG can be detected in adjacent brain tissues by MRS, gliomas can be evaluated serially even after surgical removal of a majority of the tumor. Other metabolites such as Cho, Gln, Glu, lactate, NAA and Cr can be detected in conjunction with 2HG, and this metabolic profile may be utilized to characterize tumor aggressiveness after chemotherapy and radiotherapy, at relapse and may even predict outcome [39].

Potent inhibitors of mutant IDH1 have been developed and are implicated in clinical trials in the United States. *In vitro* studies analyzing 2HG have shown a reduction of 2HG after usage of these inhibitors [40-42]. 2HG analysis by MRS would be an appropriate method to determine biological response of this drug in glioma patients.

## Conclusions

Increasing evidence suggests that 2HG is an important oncometabolite in mutant *IDH* gliomas. *In vivo* MRS has been shown to effectively measure 2HG and predict IDH status preoperatively in WHO grade II and grade III glioma patients. We found 2HG to be a positive prognostic factor in these gliomas. Further studies are warranted for other possible mechanisms of 2HG accumulation in gliomas.

## Additional file

Additional file 1:
**No survival difference between mutant** ***IDH*** **glioma patients with high vs. low 2HG accumulation.** No difference in survival between mutant *IDH* glioma patients with high 2HG accumulation (2HG >5.077 mM) vs. low 2HG accumulation was noted (p =0.8815). Median survival has not been reached in either group.

## Abbreviations

2HG: 2-hydroxyglutarate
2-HGDH: 2-hydroxyglutarate dehydrogenase
3 T: 3 tesla
α-ketoglutarate: α-KG
Cho: Choline
COSY: 2D correlation spectroscopy
Cr: Creatine
DNA: Deoxyribonucleic acid
GABA: γ- aminobutyric acid
Gln: Glutamine, Glu, glutamate
Glx: Glutamine and glutamate
GPC: Glycerophosphocholine
GSH: Gluthathione
IDH: Isocitrate dehydrogenase
IHC: Immunohistochemistry
Ins: Myo-inositol
MRI: Magnetic resonance imaging
MRS: Magnetic resonance spectroscopy
NAA: N-acetylaspartate
NAAG: N-acetylaspartylglutamate
PC: Phosphocholine
PCr: Phosphocreatine
PRESS: Point-resolved spectroscopic sequence
ROC: Receiver operating characteristic
ROS: Reactive oxygen species
SD: Standard of deviation
S/R: Signal-to-noise ratio
SVMRS: Single voxel MRS
TCGA: The Cancer Genome Atlas
tCho: Total choline, tCr, total creatine
TET: Ten-eleven translocation
tNAA: Total NAA
VLB: Volume-localized basis
VOI: Volume of interest
WHO: World Health Organization
