# Supplementary figures and images for: Accumulation of 2-hydroxyglutarate in gliomas correlates with survival: a study by 3.0-tesla magnetic resonance spectroscopy
Source: Acta Neuropathol Commun. 2014 Nov 7;2:158. doi: 10.1186/s40478-014-0158-y (PMC4236810; doi:10.1186/s40478-014-0158-y)

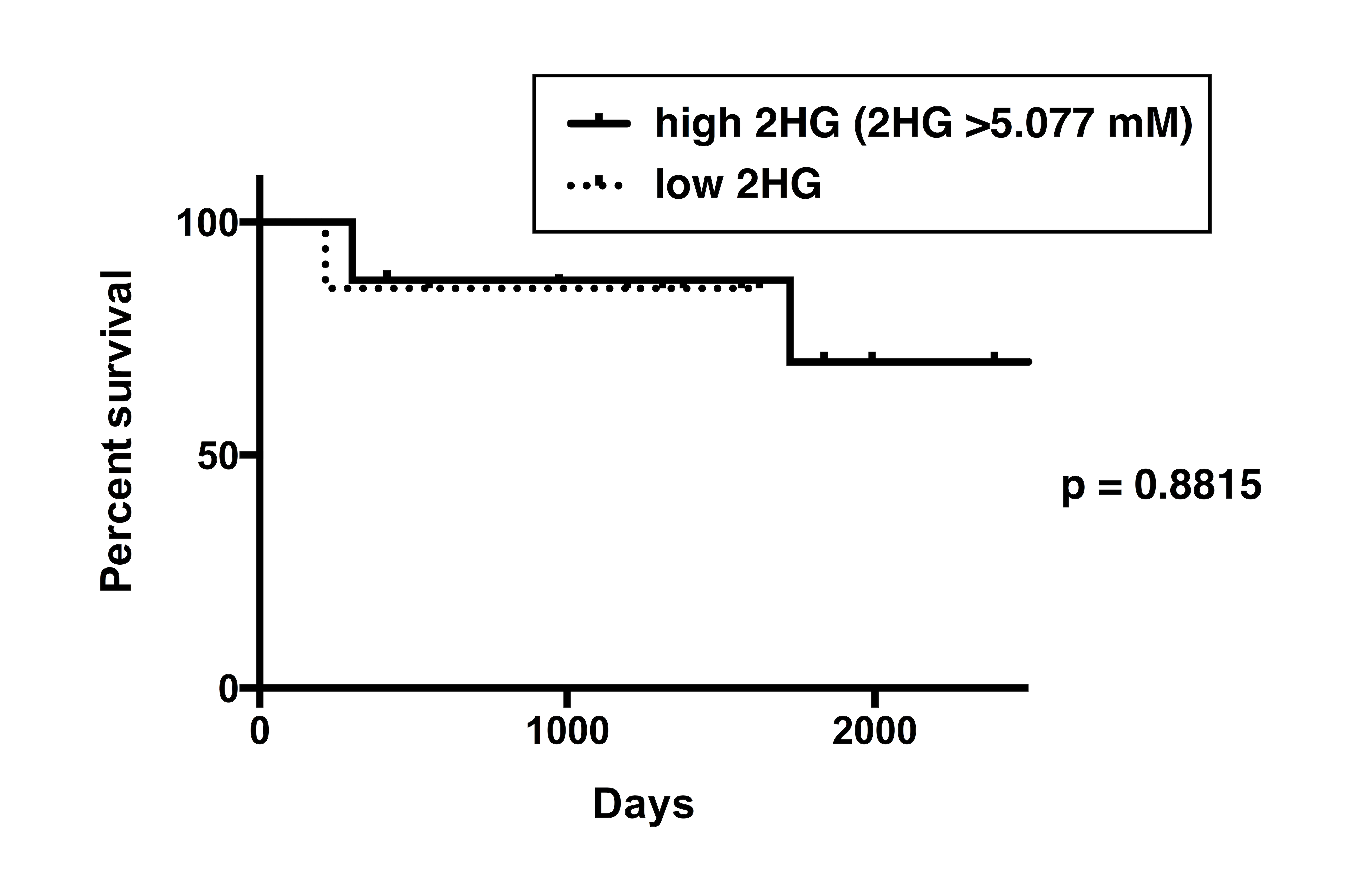

Supplement: Additional file 1: — No survival difference between mutant IDH glioma patients with high vs. low 2HG accumulation. No difference in survival between mutant IDH glioma patients with high 2HG accumulation (2HG >5.077 mM) vs. low 2HG accumulation was noted (p =0.8815). Median survival has not been reached in either group. [file 40478_2014_158_MOESM1_ESM.jpeg]
